# Redefining endpoints in heart failure clinical trials: the emerging role of wearable technologies in contemporary trial design

**DOI:** 10.1007/s10741-025-10577-0

**Published:** 2025-11-25

**Authors:** Zahra Alawoad, Jesper Jensen, Morten Schou, Nouman Ahmed, Abdelrahman Attia, Shishir Rao, Kazem Rahimi, Malgorzata Wamil

**Affiliations:** 1 Green Templeton College, University of Oxford, Oxford, UK; 2 Department of Cardiology, Copenhagen University Hospital – Herlev and Gentofte Hospital, Copenhagen, Denmark; 3 Department of Clinical Medicine, University of Copenhagen, Copenhagen, Denmark; 4 Deep Medicine, Nuffield Department of Women’s and Reproductive Health, University of Oxford, Oxford, UK; 5 Great Western Hospital NHS Trust, Marlborough Road, Swindon, UK; 6 Mayo Clinic Healthcare, 15 Portland Place, London, UK

**Keywords:** Heart failure (HF), Randomised controlled trials (RCT), Wearable devices, Patient-reported outcome measures (PROMs), Endpoints

## Abstract

Randomised controlled trials (RCTs) in heart failure (HF) have progressively broadened their primary endpoints over recent decades. Early landmark HF trials demonstrated the life-saving effects of new therapies using all-cause mortality as the definitive endpoint. As HF therapies improved survival, trial designers incorporated additional endpoints such as HF hospitalisations and quality of life. Most recently, advances in digital health have introduced wearable devices for collecting digital endpoints, enabling continuous monitoring of patient activity and physiology. This review critically examines the evolution of HF trial endpoints from a sole focus on mortality alone to modern composite and patient-reported outcomes and discusses the current challenges and opportunities of using wearable-derived endpoints in HF RCTs. Finally, we consider future directions for HF trial methodology, including regulatory and methodological considerations for integrating novel digitally collected endpoints alongside traditional measures to enhance a broad evaluation of new therapies.

## Introduction

Randomised controlled trials (RCTs) are widely regarded as the gold standard for evaluating the efficacy and safety of therapeutic interventions, forming the cornerstone of evidence-based clinical guidelines in the field of heart failure (HF). Over recent decades, HF trials have evolved in terms of trial design, outcome selection, and methodological rigour. These changes have mirrored the advancements in our understanding of distinct HF phenotypes and the growing emphasis on personalised approaches in HF management [1, 2]. However, a persistent limitation of many landmark HF trials is their reliance on highly selected patient populations, which often do not reflect the demographic and clinical diversity seen in routine practice. This discrepancy raises concerns regarding the generalisability and external validity of trial findings [3]. In response, there is increasing interest in trial designs that are more inclusive and representative.

Concurrently, the advent of wearable technologies has introduced novel opportunities for the continuous acquisition of real-world physiological data, extending beyond the temporal and spatial constraints of conventional clinic-based follow-up. These tools not only enhance the sensitivity of outcome detection and support more patient-centred assessments but also offer the potential to integrate clinical trials more seamlessly into the daily lives of individuals with HF. This facilitates the generation of dynamic, ecologically valid insights into patient status and treatment response that were previously unattainable through traditional trial methodologies dependent on intermittent, site-based evaluations. Nonetheless, the deployment of wearables may inadvertently exacerbate exclusion, particularly among older individuals or those with limited digital literacy or access. Without deliberate strategies to ensure equitable implementation, their use risks reinforcing pre-existing selection biases and widening disparities in clinical research participation.

This review critically examines the historical trajectory of choosing primary endpoints in HF RCTs, evaluates the transformative role and potential of wearable-derived digital endpoints, and discusses both the opportunities and substantial methodological challenges associated with integrating these technologies into future HF clinical research.

## Considerations for choosing a primary outcome in HF trials

The selection of appropriate endpoints is pivotal in HF RCTs, as endpoints define how therapeutic benefits are measured and interpreted. The choice must align with the clinical question under investigation and the specific phase of the study [4]. Ideally, the chosen endpoint should be directly relevant to the therapeutic intervention’s anticipated impact, reflecting outcomes meaningful to patients, clinicians, and healthcare systems alike. It is essential that the primary outcome is sensitive enough to capture the expected therapeutic effects and is anticipated to be significantly influenced by the intervention being studied. A crucial factor for endpoint suitability is the expected event rate: endpoints must occur frequently enough within the study population to ensure the statistical robustness and feasibility of the trial.

Historically, HF carried a grave prognosis – in the pre-1990s era, annual mortality rates were exceedingly high, driving a focus on survival in clinical trials [5, 6]. Early HF RCTs such as the Cooperative North Scandinavian Enalapril Survival Study (CONSENSUS) [7] and Studies of Left Ventricular Dysfunction (SOLVD) [8] exemplified this mortality-centric approach, using all-cause mortality as the primary outcome to demonstrate the life-prolonging effects of angiotensin-converting enzyme inhibitors and beta-blockers. These trials established mortality reduction as the gold-standard benchmark for HF therapy efficacy, reflecting the urgent clinical need to reduce deaths in HF patients.

Due to the singular drug development focus on reducing heavy HF-related mortality, widespread adoption of guideline-directed therapies has gradually lowered HF mortality rates, particularly in HF with reduced ejection fraction (HFrEF) [6]. As mortality rates declined, it became clear that focusing only on survival neglects HF’s broader impact on patients. The U.S. Food and Drug Administration (FDA) has evolved its perspective, shifting from requiring all-cause mortality as a primary endpoint in HF trials to emphasising cardiovascular (CV) death, reflecting the specific impact of therapies on CV outcomes [9, 10]. HF began to be recognised as a chronic illness in which morbidity, recurrent hospitalisations, symptom burden, and functional limitations play a major role in patient well-being and healthcare utilisation. Consequently, the paradigm for HF trial endpoints expanded beyond survival alone. Researchers and regulators acknowledged that exclusive reliance on mortality could underestimate the full spectrum of benefits of therapy [11]. This realisation prompted the incorporation of additional endpoints into an omnibus “composite endpoint” that captures non-fatal but clinically significant outcomes, laying the groundwork for composite and patient-centred measures in HF trials [12].

For example, the EMPHASIS-HF (Eplerenone in Mild Patients Hospitalisation and Survival Study in HF) trial used a primary composite of CV death or HF hospitalisation in patients with mild symptoms defined as New York Heart Association (NYHA) class II, demonstrating that adding eplerenone reduced this composite [13]. The inclusion of HF hospitalisations as an endpoint acknowledged the importance of lowering acute decompensation events that carry high morbidity, cost, and impact on quality of life. Composite endpoints increased event rates, improving statistical power to detect treatment effects beyond what mortality alone could show. Trials like PARADIGM-HF (Prospective comparison of ARNI with ACEI to Determine Impact on Global Mortality and morbidity in HF) similarly adopted a composite primary outcome (CV death or HF hospitalisation), reflecting a shift toward capturing both the lifesaving and morbidity-reducing effects of therapy [14]. Notably, recent drug trials (e.g., Dapagliflozin and Prevention of Adverse Outcomes in HF (DAPA-HF), Empagliflozin Outcome Trial in Patients with Chronic HFrEF (EMPEROR-Reduced) [15, 16] further refined composites by including worsening HF episodes managed in outpatient settings (requiring intravenous diuretics) as part of the primary endpoints. This broadened definition recognised that events not involving hospitalisation are clinically relevant to HF patients and can be mitigated by effective therapy.

Composite endpoints, although helpful in increasing statistical power, have limitations. Their interpretation can be complex, especially if a treatment has a disparate impact on the components [17]. For instance, as seen in the recent EMPEROR-Preserved and DELIVER (Dapagliflozin Evaluation to Improve the LIVEs of Patients with Preserved Ejection Fraction Heart Failure) trials in patients with HF with preserved ejection fraction (HFpEF) [18, 19], a composite result might be driven predominantly by reductions in so called ‘softer endpoints’ like hospitalisations, with no significant effect on mortality. In such cases, the clinical significance of the composite benefit may be debated. There is also the issue of how composite events are counted: most HF trials use a time-to-first-event analysis. However, while adding analytical complexity, recurrent events such as recurrent HF hospitalisations can be included in the primary endpoint to increase statistical power further and capture the full effect of an intervention on morbidity, as recently demonstrated in the FINEARTS-HF (Finerenone in Heart Failure with Preserved Ejection Fraction) trial [20]. Despite these nuances, the composite of CV mortality and HF hospitalisation has become *de facto* primary endpoint in modern HF trials, endorsed by regulatory bodies as a balanced measure of efficacy in a disease where therapies are expected to improve both longevity and quality of life with the disease.

## Patient-centred outcomes

Alongside mortality and hospitalisations, HF trials have increasingly incorporated endpoints that reflect symptom relief, functional capacity, and quality of life, as seen, for example, in the DEFINE-HF (Dapagliflozin Effects on Biomarkers, Symptoms and Functional Status in HF) [21] and STEP-HFpEF (Semaglutide Treatment Effect in People with Obesity and HFpEF) [22] trials. This evolution was driven by the recognition that, particularly in chronic HF or phenotypes like HFpEF, improvements in day-to-day well-being are paramount, even if mortality benefits are small [23]. This evolution reflected the growing recognition that living longer with HF must also mean living better. Patient-reported outcome measures (PROMS), such as the Kansas City Cardiomyopathy Questionnaire (KCCQ), have become standard secondary endpoints for quantifying health status from the patient’s perspective. For example, HFpEF trials (DELIVER [19], EMPEROR-Preserved [18]) included changes in KCCQ summary scores as secondary or at least prespecified endpoints alongside traditional composite outcomes, acknowledging that symptom burden is a central concern in this cohort.

## Functional outcomes

Objective functional measures, such as the 6-minute walk test (6MWT) or peak oxygen uptake (VO₂), have also been used, particularly in trials evaluating interventions aimed at improving exercise capacity (e.g., rehabilitation or mechanical circulatory support studies). In PARADIGM-HF, for instance, the angiotensin–neprilysin inhibitor showed improvements in KCCQ scores, reflecting better quality of life, in addition to reducing CV death and HF admissions [14]. Such findings provide a more holistic view of benefits and can inform shared decision-making, aligning trial outcomes with what matters to the patient. Nonetheless, challenges remain in using these endpoints: PROMs introduce a level of subjectivity and functional tests can be effort-dependent, whereas RCTs must ensure randomisation, blinding and accurate ascertainment of outcomes to minimise bias and ensure the validity and reliability of trial findings. Despite these challenges, the trend toward patient-centric endpoints marks a positive shift in HF research, broadening the definition of the benefit of therapy [24].

## Wearable device-derived endpoints

**Fig. 1:**
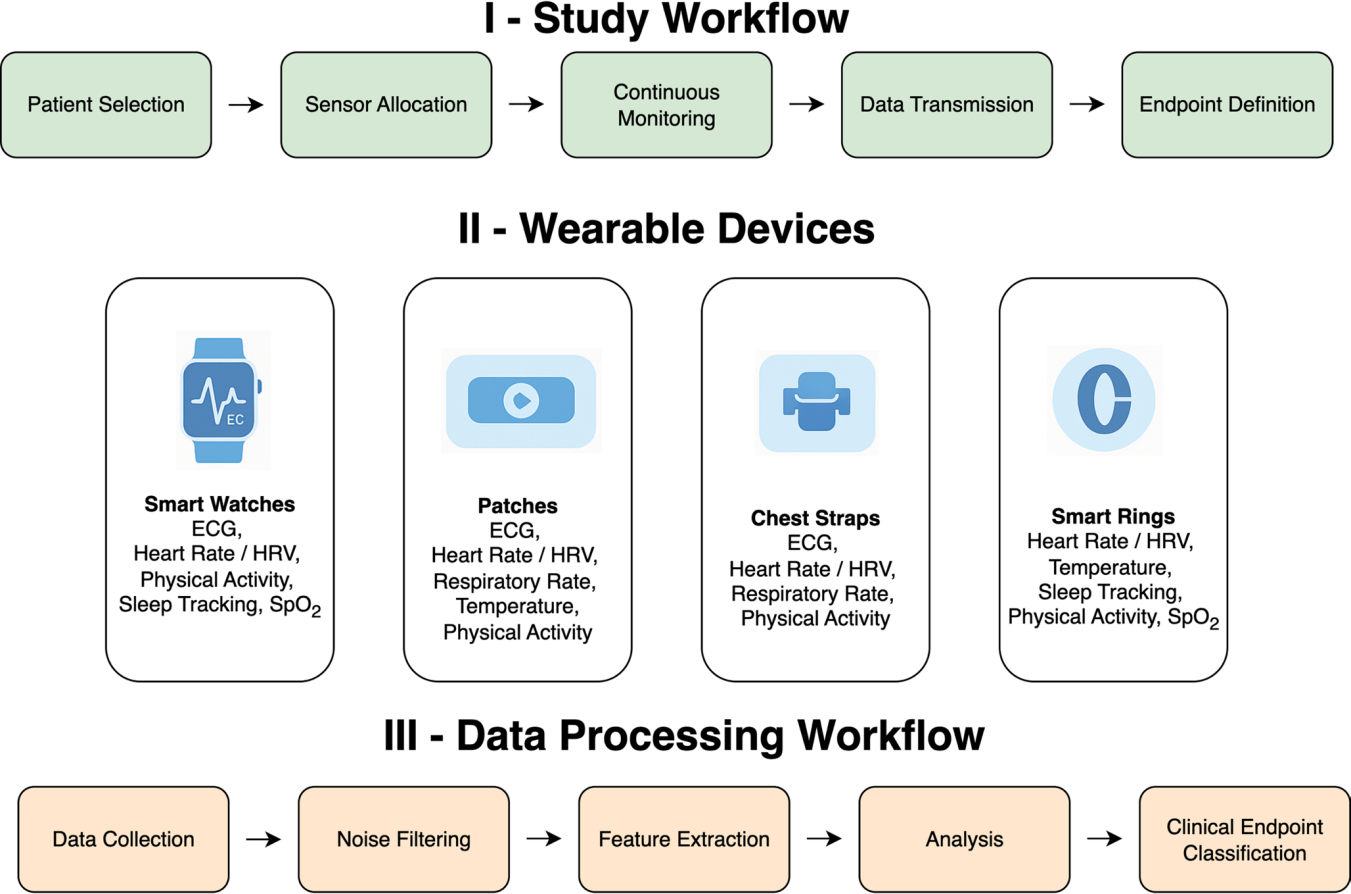
Overview of Wearable Integration in Randomised Controlled Trials (RCTs). The figure illustrates a generalised workflow for incorporating wearable technology in RCTs, encompassing (I) the study workflow from patient selection to endpoint definition, (II) examples of common wearable devices and their collected physiological data, and (III) the data processing workflow from initial data collection to clinical endpoint classification

The rapid advancement of wearable technology and digital health drives the latest phase in the evolution of HF trial endpoints. In Table 1, examples of wearable-derived endpoints in HF populations are summarised, indicating the diversity of wearables and objectives. Wrist-worn trackers, ranging from activity trackers (dedicated fitness bands and pedometers) and smartwatches to patch-based sensors, can continuously and remotely monitor various physiologic and behavioural parameters of HF patients in their everyday environments (Fig. 1). This capability opens new possibilities for endpoint assessment in HF RCTs, as investigators can now capture patient activity levels, heart rate and rhythm, blood pressure, sleep patterns, and even cardiopulmonary metrics in real-time. Wearable sensors thus enable a shift from intermittent, clinic-based measurements to continuous monitoring, potentially providing a more granular and holistic view of how HF patients respond to therapy. For example, rather than relying only on clinic assessments of functional class, such as the NYHA classification, a trial could use daily step count or physical activity duration (derived from accelerometers) as an endpoint to quantify improvement in a patient’s functional capacity in real-world settings, as has been done with several different accelerometer devices (see examples in Table 1). Early studies have demonstrated that such wearable metrics correlate with traditional functional measures: in the recent DETERMINE-HF and EMPIRE-HF trials, baseline daily activity level tracked by an accelerometer showed a modest correlation with 6MWT distance and KCCQ scores [33–35]. Interestingly, changes in activity did not perfectly mirror changes in 6MWT or KCCQ, suggesting that wearable data provide complementary information about patient status [33]. This indicates that wearable-derived endpoints could enrich our understanding of treatment effects by capturing aspects of patient function that clinic tests and questionnaires might miss (for instance, spontaneous daily mobility vs. structured test performance).

**Table 1:** Examples of wearable-derived endpoints relevant to HF trials

Study	Year	Wearable type	Number of participants	Objectives	Results
EVANGELISTA, et al. [25]	2005	Hip-borne pedometer (Sportline Pedometer Model 330)	38	Determine if pedometers offered a way to measure exercise adherence to a home-based walking program among HF patients and their effect on functional status.	Adherers demonstrated higher distance walked by pedometer than non-adherers by 3 and 6 months. Significant correlation between adherence and measures of functional status (measured by 6MWT and VO2 max)
BURCH et al. [26]	2020	The WCD LifeVest (ZOLL)	197	Evaluate the accuracy and reliability of the wearable cardioverter defibrillator-guided 6MWT performed at home by patients with HF versus in-clinic testing.	The results of the in-clinic 6MWT were similar between clinician-guided and WCD-guided patients across all objective distances. Distances walked with a WCD-guided walk test were consistent whether conducted in the clinic or at home and were reliable over time.
Dorsch et al. [27]	2021	ManageHF4Life app + Physical activity monitor (Fitbit Charge 2) and scale (Fitbit Aria and Aria 2)	83	Evaluate the efficacy of enhanced self-management via a mobile app intervention on health-related quality of life, self-management, and HF readmissions	The adaptive mobile app intervention improved the MLHFQ at 6 weeks but did not sustain its effects at 12 weeks. No effect was seen on HF self-management measured by self-report
Nagatomi et al. [28]	2022	Fitbit wrist device + App	30	Investigate the efficacy and safety of a comprehensive home-based cardiac rehabilitation (HBCR) program using information and communication technology (ICT).	A comprehensive HBCR program using ICT for HF patients with physical frailty improved exercise tolerance and improved lower extremity muscle strength
Butler et al. [29]	2023	The ActiGraph GT9X Link accelerometer	69	Describe the activity profile of patients with HF with preserved ejection fraction (HFpEF) acquired during accelerometry recordings and relate it to changes in KCCQ-PLS and 6MWT from baseline to Week 24	Accelerometer-based activity measures did not correlate with subjective or objective standard measures of health status and functional capacity in heart failure with preserved ejection fraction
Dibben et al. [30]	2023	GENEActiv triaxial accelerometer	173	Quantify the impact of a home-based cardiac rehabilitation intervention on objectively assessed physical activity of patients with heart failure and explore the extent by which patient characteristics are associated with a change in PA	Home-based cardiac rehabilitation intervention did not increase overall weekly activity; patients’ behaviour patterns appeared to change with increased weekday PA levels and reduced inactivity.
Vetrovsky et al. [31]	2023	Actigraph wGT3X-BT accelerometer	202	Determine whether a 6-month lifestyle walking intervention improves functional capacity assessed by the 6MWT in patients with stable heart failure with reduced ejection fraction compared with usual care.	Lifestyle intervention improved daily steps by about 25%. No improvement in functional capacity
Alvarez-Garcia et al. [32]	2024	Remote dielectric sensing (ReDS) system	100	Assess whether the ReDS-guided strategy during acutely decompensated HF hospitalization is superior to routine care for improving outcomes at 1-month post-discharge. **Primary outcome**: composite of unplanned visits, rehospitalisation, or death at 1 month after discharge.	Reduction in the 30-day primary composite outcome mainly because of a decrease in the number of HF readmissions

*6MWT* Six-Minute walk Test, *VO2 max* peak oxygen uptake, *HF* heart failure, *MLHFQ* Minnesota living with heart failure Questionnaire, *KCCQ-PLS* Kansas City cardiomyopathy Questionnaire-Physical limitation Score, *PA* Physical activity, *ReDS* The remote dielectric sensing

Wearables can also detect clinically important events that might otherwise go unreported between trial visits. A pertinent example in CV research is the use of smartwatch photoplethysmography to intermittently screen for atrial fibrillation (AF), as evidenced by the Apple Heart Study, which, in a virtual, app-based study, enrolled over 400,000 participants nationwide and successfully identified new AF cases (*n* = 153 new AF cases) [36]. In HF, continuous wearables monitoring could similarly detect subclinical fluid retention episodes or arrhythmias that portend HF decompensation; however, testing in large-scale clinical trials is needed to establish the clinical utility of such continuous monitoring [37, 38]. As an illustration, a multi-sensor wearable could track a patient’s resting heart rate, respiratory rate, and activity; an upward drift in resting heart rate coupled with reduced daily steps might signal worsening HF before the patient requires urgent care. Although invasive, devices such as the CardioMEMS system offer proof of principle that proactive detection of fluid retention can significantly impact patient management and a similar concept could be utilised in the wearables field. The CardioMEMS device measures pulmonary artery pressure directly and transmits data remotely to healthcare providers, allowing them to detect fluid overload early and intervene before clinical symptoms emerge or worsen [39, 40]. As a non-invasive alternative to CARDIOMEMS, the CardioTag system is a recent wearable device that may be used to measure right-sided heart pressures, potentially increasing accessibility and improving cost-effectiveness of hemodynamic-guided HF-management [41]. Capturing such dynamic endpoints (e.g., day-to-day variability in physiological markers or activity) enables a more nuanced evaluation of how an intervention stabilises HF trajectory or reduces volatility in a patient’s condition.

The extensive datasets generated by wearable devices constitute a promising resource within healthcare research, particularly when harnessed through artificial intelligence (AI) and machine learning (ML). These novel methodologies enable sophisticated predictive modelling, transforming continuous physiological data streams into actionable clinical insights. ML is uniquely suited to leverage these rich, dynamic datasets, facilitating the identification of subtle patterns and predictive signatures that conventional statistical methodologies might overlook [42]. Consequently, ML-driven analysis allows for nuanced risk stratification, precise phenotypic characterisation of diseases, and detailed mapping of disease trajectories. Thus, the predictive modelling capabilities inherent in ML can potentially redefine the traditional approach to primary outcomes in clinical trials, enhancing the sensitivity and accuracy of therapeutic efficacy assessments and offering novel perspectives on disease progression and patient responsiveness to interventions. For example, in large clinical trials where data collection has included continuous monitoring with a wearable device, the application of data analysis with AI and ML may potentially identify both new phenotypes of patients, new wearable endpoints, and differences in responsiveness to an intervention at different stages of disease.

Another promising aspect of wearables is their potential to facilitate decentralised trial designs. By allowing remote data capture, wearables reduce the reliance on frequent in-person visits for endpoint assessment. This can ease participant burden and improve accessibility, particularly for individuals with limited mobility or those residing far from research centres. Digital health initiatives, such as the MyHeart Counts study, have demonstrated the feasibility of remote recruitment and data collection at scale [43]. In HF RCTs, such approaches may enhance the inclusion of underrepresented populations and enable more pragmatic trial designs. Moreover, continuous out-of-clinic monitoring mitigates recall bias and yields objective, real-time data, improving dataset completeness and capturing clinically relevant changes that might otherwise go undetected.

Therefore, wearable-derived endpoints offer several opportunities: more patient-centred assessments, more sensitive detection of changes or events, and more inclusive trial conduct through remote participation.

## Limitations of utilising digitally derived endpoints in HF trials

Despite their potential, wearable-derived endpoints in HF research face significant regulatory challenges, with implications for investigators at all stages of development, from endpoint selection, over technical and clinical validation, to evaluation of the candidate digital endpoint (Table 2). Regulatory bodies require rigorous validation to establish that such endpoints are clinically meaningful and reliable. Agencies such as the FDA and the European Medicines Agency demand evidence that digital measures correlate with established outcomes and reflect meaningful clinical benefit. In HF, no wearable metric has yet reached this level of acceptance for use as a primary endpoint in a phase III RCT. Although regulators have issued draft guidance supporting digital health technologies, they remain cautious [44]. To gain acceptance, trialists must engage early with regulators and adhere to frameworks such as the Clinical Trials Transformation Initiative (CTTI) to ensure data quality and regulatory alignment [44].

**Table 2:** Selecting efficacy endpoint in a pharmacological multicentre randomised controlled trial using wearable sensors

Stage of validation of a digital endpoint	Factors to consider	Investigative focus
Endpoint selection	Specific objectives of the trial Characteristics of the drug being tested Condition being treated	Identification of key metrics relevant to the target population.
Technical validation	Comparison of the chosen technology against standard way of measuring outcome Assessment of user experience and efficiency of data transfer Sensitivity and specificity of detecting an endpoint	Accuracy/validity vs. gold standard method Precision/repeatability, sampling frequency/latency Robustness across conditions Data integrity: loss rates, encryption, transfer reliability
Clinical validation	Correlation with traditional outcome measures Useability and compliance Difference between control subjects and the studied population Evaluation of endpoint consistency across various research centres Consistency across patient demographic stratifications (e.g. sex, skin tone)	Identification of the wearable sensor’s relevance to the drug’s therapeutic effects and the condition being treated Confirmation of the validity of the digital endpoint in the studied population
Candidate digital endpoint	Effect size and sample size for target population Feedback from patients, doctors, and regulators	Evaluation of the device’s additive value within the clinical trial Assessment of the device’s capability to detect the treatment effect

Another critical challenge lies in the methodological and data considerations of using wearables (Fig. 1). These devices generate continuous streams that are high volume, high frequency, and multidimensional and require substantial infrastructure and analytic expertise [45]. For example, investigators must pre-specify and validate how to distil meaningful endpoints from the raw data (defining an outcome as the average daily steps over a 2-week period at the trial end). Inappropriate analysis could inflate type I errors or yield spurious correlations. Moreover, missing data is a concern: devices may fail, or participants might not wear them consistently, leading to gaps [46]. As such, wearable-derived continuous monitoring may introduce higher standards for data completeness, opening the door for more missing data. Therefore, ensuring device reliability and patient adherence is essential. Technical malfunctions can compromise data integrity, and complex devices can overwhelm patients, especially HF patients who may not be tech-savvy. Low adherence in wearing or charging the device can result in significant data loss, reducing trial power or introducing biased results if non-adherent participants differ systematically from adherent ones. Trials must incorporate robust training, user-friendly device design, and data feed monitoring to identify and rectify device issues quickly. Some studies provide technical support or even employ run-in periods to ensure participants can use the wearable as intended. Early and continuous collaboration with the device manufacturer is key for the correct use of the device and complete data collection, which ultimately ensures the reliability of the wearable-derived endpoint.

Data privacy and security present additional challenges. Wearables continuously collect sensitive personal health data, raising concerns about how that data is stored, transmitted, and protected [47]. Multicentre HF trials often span regions with different privacy laws. Investigators must implement strict data encryption, secure servers, and clear consent processes informing participants about what data is collected and who will access it. Engaging third-party tech companies (e.g. cloud platforms or device manufacturers) introduces potential vulnerabilities, so agreements must delineate data ownership and confidentiality responsibilities [48]. Ethical oversight committees increasingly request detailed data management and privacy protection plans when wearables are used. Thus, maintaining data integrity and participant trust is as important as the scientific aspects of wearable integration.

Another consideration is equity and generalisability. The so-called ‘digital divide’, which defines the gap in technology access and literacy, means that reliance on wearables could inadvertently exclude or under-serve certain populations [49]. Older HF patients, rural residents, or those from lower socioeconomic backgrounds, who are disproportionately affected by HF, may be less likely to own or use sophisticated wearable devices [50]. If a trial requires a smartphone app or wearable sensor, patients without compatible phones or limited internet access might be unable to participate, and this can lead to selection bias. Ensuring that RCTs utilising wearables remain inclusive may require providing devices and data plans to participants who need them, simplifying device interfaces, and offering technical support and education. Sensitivity analyses are also required to detect any differential effects among participants, for example, whether the intervention’s benefit on a wearable-derived endpoint applies equally in older vs. younger patients, to ensure that digital endpoints are valid across the broad HF population.

Finally, there is the issue of clinical interpretability of wearable endpoints. Even if a trial shows a statistically significant improvement in a digital measure, defining the minimal clinically important difference for a novel endpoint (e.g. how many more steps per day equate to a perceptible improvement in exercise tolerance?) is not straightforward [51]. Such thresholds often require anchoring the new metric to established outcomes, for instance, correlating a change in wearable-measured activity with changes in 6MWT distance or NYHA class. Until a new endpoint is validated in this way, its standalone interpretation may remain abstract. Caution is warranted that enthusiasm for technology is balanced with scientific rigour and patient safety at every step of trial design. Ongoing collaboration among academia, industry, and regulators (for instance, through public-private partnerships and consortia focused on digital endpoints) will be crucial to establish standards for device validation, data quality, and clinical significance.

## Future perspective

Advancements in HF research increasingly suggest the adoption of hybrid endpoint frameworks that incorporate traditional clinical outcomes alongside patient-reported and sensor-derived metrics [46]. Such composite endpoints could, for instance, integrate traditional clinical measures like HF hospitalisation with patient-centred outcomes, including meaningful symptom improvements measured by questionnaires such as the KCCQ or sensor-based assessments of daily activity levels. Employing these multi-domain endpoints enables a more holistic evaluation of therapeutic interventions, capturing not only clinical events but also patient-centred perspectives and continuous physiological data. As clinical trials evolve, the adoption of these hybrid models is likely to become prevalent, reflecting a nuanced approach to assessing disease progression, patient experience, and intervention impact in HF research.

Technological progress will likely make wearables smaller, more comfortable, and more capable, further blurring the line between clinical monitoring and everyday life. Emerging devices and algorithms could enable the detection of subtle HF decompensation signals (like changes in thoracic impedance or respiratory rate) and trigger pre-emptive interventions, making the RCTs safer. Future HF RCTs might test dynamic interventions incorporating an adaptive design where early warning from wearable sensors prompts protocolised adjustments (such as medication up-titration). Moreover, integrating AI within such interventions fed by wearable data holds promise: ML models can mine continuous data for patterns predictive of outcomes, potentially identifying responders to therapy or personalising endpoints for individual patients. While such applications are nascent, early studies have demonstrated feasibility (e.g. AI analysis of smartwatch ECGs to detect low ejection fraction) [52].

In conclusion, the endpoints used in HF RCTs have evolved from focusing narrowly on mortality to encompassing the multifaceted goals of therapy, prolonging life, reducing hospital admissions, improving functional capacity, and enhancing quality of life. The advent of wearable technology offers a compelling new dimension to this evolution. Wearable-derived endpoints have the potential to make HF trials more patient-centred. Still, they also introduce validation, data handling, and interpretation challenges that the research community must carefully address. A rigorous, critical approach is needed to integrate wearables into HF RCTs, leveraging their strengths while mitigating their limitations. As we refine these tools and accumulate evidence, it is foreseeable that future HF trials will use a mix of traditional and wearable-derived endpoints to provide the most comprehensive assessment of new therapies. This balanced approach, rooted in scientific rigour and centred on patient well-being, will ensure that the next generation of HF trials continues to deliver findings that meaningfully inform clinical practice and ultimately improve outcomes.

## Data Availability

No datasets were generated or analysed during the current study.
